# Two Thousand Prescriptions Analysed

**Published:** 1909-04-17

**Authors:** 


### THERAPEUTICS AND PHARMACY.

### TWO THOUSAND PRESCRIPTIONS ANALYSED.

It is always of interest to medical men to learn what pharmacists have to say about the prescriptions that they receive. A paper upon 2,000 consecutive prescriptions received and dispensed was read recently before the Manchester Pharmaceutical Association by Mr. J. Woodruff Walton and printed in a recent number of the *Pharmaceutical Journal*. The following points are abstracted from it:—

In the first place, contrary to what might have been expected, it is remarkable how few times proprietary articles that are so much "puffed" and advertised were ordered by the medical profession amongst the entire two thousand prescriptions. Next, mixtures are still by far the most common form of administration; altogether 1,359 different mixtures were ordered. The number of ingredients in these reveal how various are the customs of different prescribers. Some medical men rely on one or two ingredients in their mixtures, whilst others crowd a multiplicity of preparations into a bottle, and often the resulting mixture became so complicated that the effects of some of the drugs must have been counteracted by those of others.

Without counting aqua destillata as a British Pharmacopœial preparation, it was found that—

| | | | | |
|---|---|---|---|---|
| 100 | of the mixtures | contained | 1 | ingredient each. |
| 200 | " | " | 2 | " |
| 330 | " | " | 3 | " |
| 369 | " | " | 4 | " |
| 223 | " | " | 5 | " |
| 98 | " | " | 6 | " |
| 31 | " | " | 7 | " |
| 6 | " | " | 8 | " |
| 2 | " | " | 9 | " |
| 1,359 | | | | |

The two mixtures that contained nine preparations each may be given in full, for it will be seen that each really contained a still larger number of substances than at first sight appears. The first contained:

Pot. Bicarb.; Oxymel. Scillæ; Tinct. Camph. Co.; Tinct. Gent. Co.; Mucil. Acac.; Spt. Chlorof.; Tinct. Lobeliæ Æth.; Glycerin.; Aq. Menth. Pip. ad ℥viij.

If this mixture is analysed into its actual contents, exclusive of the menstruum such as water or spirit, it will be found that it contains—

Pot. Bicarb.

Squills, Acetic Acid, Clarified Honey } Oxymel Scillæ.

Tincture of Opium, Benzoic Acid, Camphor, Oil of Anise } Tr. Camph. Co.

Gentian Root, Bitter Orange Peel, Cardamom Seeds } Tr. Gent. Co.

Gum Acacia

Chloroform

Lobelia

Æther

Glycerin

Oil of Peppermint; making a total of seventeen drugs in one mixture.

The other example contained—

Bismuth. Salicyl.; Bismuth. Carb.; Liq. Opii Sed. Battley; Tinct. Nuc. Vom.; Ext. Cascar. Sagr. Liq.; Tinct. Chlorof. Co. 1885; Tinct. Belladonnæ; Dec. Aloes Co.; Inf. Gent. Co. ad ℥viij.

This is a very complicated mixture, as is shown by analysing it into its constituents:—

Opium, Sherry } In the Liq. Opii Sed. whose formula is not definitely known.

Nux Vomica

Cascara

Chloroform, Cardamom Seeds, Caraway Fruit, Raisins, Cinnamon Bark, Cochineal } Tr. Chlorof. Co.

Bismuth. Salicylate

Bismuth. Carbonate

Belladonna

Aloes, Myrrh, Saffron, Potassium Carbonate, Liquorice } Dec. Aloes Co.

Gentian Root, Bitter Orange Peel, Fresh Lemon Peel } Inf. Gent. Co.

The next most numerous prescriptions were powders, of which there were 125, and ointments 124. It appears that cachets are coming into vogue more and more, and that pills are on the decline. The former were ordered in 63 cases; the latter in only 42. Lotions totalled 85, liniments 52. Tablets were prescribed 60 times; capsules, 40; gargles, 29; paints, 19; lozenges, 15; inhalations, 22. British proprietary articles and preparations were ordered 179 times; German proprietary articles, 84; American, 41; and French, 5.

One hundred and fifty-three preparations were prescribed from the British Pharmaceutical Codex. The British Pharmacopœia of 1885 still has its adherents, for on seventy-three occasions preparations from it were ordered. Of these the two most favoured were tinctura chloroformi composita and tinctura chloroformi et morphinæ.

